# Mr. Fogo's Remarks on a Case of Phthisis

**Published:** 1804-02-01

**Authors:** A. Fogo

**Affiliations:** Newcastle


### *To the Editors of the Medical and Physical Journal.*

Gentlemen,

In the Med. Journal, No. 59, p. 551, there is a Case related by Mr. Dunn, which he asserts to be phthisis pulmonalis, and that he cured it by digitalis. "E. Coulston, about five or six weeks from (I suppose he means before) the commencement of this history, had been attacked with fever of an inflammatory nature. She had been bled by a medical gentleman two or three times, and taken nitrous medicines; that an affection of the lungs took place, plainly evincing a translation of morbid matter to those organs; that blisters and nitre had been used to little purpose."

The above is an imperfect account of the origin of the disease. We are not told whether the fever was attended

 with with topical inflammation or not; nor, if there was, where it existed; whether pleuritis, or hepatitis; nor where the blisters were applied; nor from whence the morbid matter was translated to the lungs.

Mr. D. goes on to describe (he says accurately) the condition in which he found her on the 24th of June, 1803, six or seven weeks after the commencement of the disease, "A violent cough, great pain, and spasmodic action of the diaphragm, drawing in the scrobiculus cordis; a rattling noise in the throat; expectoration of a great quantity of thin matter; (was it purulent?) great emaciation, yellow countenance, hectic flushes, colliquative diarrhœa, dyspnœa; pulse quick, hard; loss of appetite, pain in lying on the left side."

I suspect the disease in the lungs to have been a secondary affair; and from the complaints about the diaphragm, scrobiculus cordis, yellow countenance, and, as he afterwards says, "the drawing in of the umbilicus, which all along had been productive of great pain and distress in coughing," I am inclined to believe the original disease to have existed in the liver, as the parts principally affected are closely connected with that organ.

He gave "gtt. ij. tinct. digital. every four hours in an oleaginous mixture with a small quantity of nitre.

June 25, gave gtt. iij. every four hours.

26. Ditto.

27. Cough considerably abated, diarrhœa almost gone, expectoration less, and thicker; feet swelled; gtt. iv. 4tis horis; cough worse in the afternoon, &c.

28. More cheerful, less fever and cough; had expectorated, since yesterday morning, a great deal of tough viscid phlegm, consisting of fibres and threads; complained of an itching in the nose and about the anus, with costiveness. He suspected worms in the intestines. Gave a purging powder.

29. The powder operated five or six times, and brought off a quantity of dark coloured fæces, and so black that if there had been worms they would have been indiscoverable; gave gt. vj. 4tis. horis.

30. Much better, all the symptoms relieved, more cheerful; slept a little, pulse about 70."

The state of the pulse now may be called a natural and healthy state, and is the first, and the only, time Mr. D. has mentioned the number of strokes in a minute through the whole of the case. From this day she recovered gradually

I mentioned

I mentioned my suspicions above that the disease originated in the liver; and, from its termination, I am inclined to think they were well founded. Whence the yellow countenance, so unlike the red and white in phthisis? Do disorders in the diaphragm attend phthisis? Was ever a phthisis pulmonalis cured or checked by 90 drops of tinct. digitalis in four days? Did ever phthisis pulmonalis cease on the discharge of a quantity of dark coloured fæces? The last mentioned circumstance requires some investigation.

Mr. D. says there existed a colliquative diarrhœa on the 25th. I wish to know where the black fæces were lodged at that time, which were discharged on the 28th? They could not be within the intestinal tube; they must have been confined in some corner without the tube; and there is no corner so likely as in the large obstructed vessels of the liver. I have attended many cases of obstructions of the vessels of the liver, and could always promise a recovery when fæces of a dark colour made their appearance. But as no notice is taken of the colour of the fæces before or after the 29th, the case is imperfect; which may be accounted for from Mr. D. viewing the disease as phthisis pulmonalis, and overlooking the cause of the principal symptoms, which pointed out to me a very different disorder.

Though I value the virtues of digitalis from the good effects I have witnessed in several cases, or rather in several constitutions, for it appears to have very different effects in the same disease in different patients, yet I do not expect that it will ever perform a miracle, such as the case above would appear to be.

If these remarks meet with your approbation, so as to be honoured with a place in your Journal, and if they should fall into the hands of the unknown gentleman who attended E. Coulston from the first, a few lines from him, describing the origin, progress, and symptoms during his attendance, would be esteemed a favour.

